# Pathogenesis and management of traumatic brain injury (TBI): role of neuroinflammation and anti-inflammatory drugs

**DOI:** 10.1007/s10787-022-01017-8

**Published:** 2022-07-08

**Authors:** Sunishtha Kalra, Rohit Malik, Govind Singh, Saurabh Bhatia, Ahmed Al-Harrasi, Syam Mohan, Mohammed Albratty, Ali Albarrati, Murtaza M. Tambuwala

**Affiliations:** 1 Department of Pharmaceutical Sciences, Maharshi Dayanand University, Rohtak, Haryana India; 2 School of Health Sciences, University of Petroleum and Energy Studies, Dehradun, Uttarakhand India; 3 Natural and Medical Sciences Research Centre, University of Nizwa, Birkat Al Mauz, Nizwa, Oman; 4 Substance Abuse and Toxicology Research Centre, Jazan University, Jazan, Saudi Arabia; 5 Department of Pharmaceutical Chemistry, College of Pharmacy, Jazan University, Jazan, Saudi Arabia; 6 Rehabilitation Health Sciences, College of Applied Medical Sciences, King Saud University, Riyadh, Saudi Arabia; 7 School of Pharmacy and Pharmaceutical Sciences, Ulster University, Northern Ireland, UK

**Keywords:** Anti-inflammatory drugs, Clinical trial, Management, Neuroinflammation, Pathogenesis, Traumatic brain injury

## Abstract

Traumatic brain injury (TBI) is an important global health concern that represents a leading cause of death and disability. It occurs due to direct impact or hit on the head caused by factors such as motor vehicles, crushes, and assaults. During the past decade, an abundance of new evidence highlighted the importance of inflammation in the secondary damage response that contributes to neurodegenerative and neurological deficits after TBI. It results in disruption of the blood–brain barrier (BBB) and initiates the release of macrophages, neutrophils, and lymphocytes at the injury site. A growing number of researchers have discovered various signalling pathways associated with the initiation and progression of inflammation. Targeting different signalling pathways (NF-κB, JAK/STAT, MAPKs, PI3K/Akt/mTOR, GSK-3, Nrf2, RhoGTPase, TGF-β1, and NLRP3) helps in the development of novel anti-inflammatory drugs in the management of TBI. Several synthetic and herbal drugs with both anti-inflammatory and neuroprotective potential showed effective results. This review summarizes different signalling pathways, associated pathologies, inflammatory mediators, pharmacological potential, current status, and challenges with anti-inflammatory drugs.

## Introduction

Traumatic brain injury (TBI) is identified as an important global health concern which represents a leading cause of death and disability. TBI occurs due to direct impact or hit on head, caused by a number of things including motor vehicles, crushes, and assaults. The result of initial mechanical damage that happens at the time of injury is referred to as a primary injury. Primary injury is responsible for initiation of secondary injury. Secondary injury develops over a periods of time after primary injury (Hovda et al. 1994; Xiong et al. 1997; Singh et al. 2006). Secondary injury cascades including oxidative stress, endoplasmic reticulum stress, and neuroinflammation contribute to long-term brain damage and can be triggered by a variety of risk factors. These damage cascades converge on an early tau acetylation route, which may act as a catalyst for subsequent degeneration (Lucke-wold et al. 2017). Onset of secondary injury is a result of physiological and biochemical cascades that finally leads to neuronal cell death and functional impairments. Primary injury is an irreversible approach because it is refractory to most therapeutic strategies. It is only prevented by use of safety devices (Werner and Engelhard 2007; Mbye et al. 2008). The interval during which secondary injury develops gives a golden chance for medical approaches that has the ability to prevent and reduce secondary harm while also improving long-term clinical outcomes. However, significant preclinical findings have yet to be validated in clinical trials. Clinical trials have failed due to the physiological variability of trauma patients, as well as a lack of comprehensive pharmacokinetic study for determining the best dosage, starting time of therapy, and therapeutic period of the target drugs (Schouten 2007). As a result, understanding of various molecular and cellular factors which leads to secondary injury is necessary for developing successful neuroprotective approaches for TBI. Neuroinflammation is a secondary damage response that contributes to neurodegenerative and neurological deficits after a TBI. Although most researchers have highlighted negative neuroinflammatory consequences on damaged brain, significant benefits can be obtained if neuroinflammation is treated in a controlled manner. We will look at synthetic and herbal anti-inflammatory drugs that have been explored as therapeutic options for TBI and have shown potential in clinical trials.

### Neuroinflammation in pathogenesis of TBI

Neuroinflammation plays an important role in neurological impairments and neurodegeneration that might occur after TBI. Increased levels of inflammatory mediators, glial cell activation, and leukocyte recruitment are signs of post-traumatic neuroinflammation (Morganti-Kossmann et al. 2007).

TBI results in disruption of BBB, initiate release of macrophages, neutrophils, and lymphocyte at site of injury. Researchers, working on TBI in animals and humans, have observed an increase in blood-borne immune cells inside the brain parenchyma. Inflammatory mediators are released by these cells, which attract immune and glia cells to the injured area. In addition to immune cell invasion, resident microglia activation plays an important role in damage. Microglial processes form a first-line defence barrier between healthy and injured area of brain (Davalos et al. 2005; Haynes et al. 2006). Whenever microglia become excessively reactive or activated, they release oxidative metabolites (e.g. nitric oxide and reactive oxygen species) as well as pro-inflammatory cytokines [e.g. tumour necrosis factor α (TNF-α), interleukin (IL-1β), and interferon γ (IFN-γ)] that have a detrimental impact on neurons (Block and Hong 2005). Furthermore, production of pro-inflammatory cytokines and supplementary components determines the successive stimulation of astrocytes and glial scar formation in brain injury. Development of intermediary filaments (GFAP and vimentin), elevated cellular accumulation, and cell swelling are all signs of astrocyte activation (Herrmann et al. 2008).

Corresponding to microglia, reactive astrocytes also produce destructive as well as neuroprotective effects in TBI. Activation of astrocyte stimulates variety of neurotrophic factors like brain-derived neurotrophic factor (BDNF) to protect and support brain from cellular death persuaded by injury (Zhao et al. 2004). Moreover, astrocytes are important regulators of extracellular glutamate level and responsible for reducing glutamate excitotoxicity in neurons as well as neuroglia (Schousboe and Waagepetersen 2005). Particularly, damaged astrocytes aggravate transgenic depletion and neuronal deterioration of reactive astrocyte which consequently promotes neuronal death and assists terrible consequences following TBI (Maeda et al. 2003). Hypertrophic astrocytes around lesion site after damage form a suppressive extracellular matrix containing chondroitin sulphate proteoglycans, which prompt the formation of glial scar. The developed strong physiochemical barrier restricts functional connections needed for axonal repair and growth, as well as impedes axonal regeneration (Cafferty et al. 2007). Contrarily, astrocytes give nutritional guidance and support throughout axonal growth after neuronal injury, but chronic astrogliosis limits and impairs functional recovery and axon regeneration (Menet et al. 2003; Wilhelmsson 2004).

### Inflammatory signalling pathways in pathogenesis of TBI

*Nuclear factor-kappa B (NF-κB)* NF-kB is an imperative inflammatory signalling pathway which involves in the synthesis of inflammatory molecules and pro-inflammatory genes such as cytokines and chemokines (Liu et al. 2017b). NF-kB is a downstream element for the stimulation of various receptors such as toll-like receptor 4 (TLR-4) and tumour necrosis factor receptor-associated factor6 (TRAF-6) in human and animals which suffered with TBI. As a consequence, inhibiting NF-kB reduces apoptosis and inflammation following injury. A previous study reported that NF-kB activation in glial and neuronal cells is associated with neuroprotective activity and neurodegenerative diseases (Singh and Singh 2020).In glial cells, NF-kB promotes inflammation, whereas in neurons it plays a role in synaptic plasticity, neuronal development, survival, and synaptic plasticity (Mattson and Camandola 2001). NF-kB levels were found to be higher in rats following fluid percussion and controlled cortical impact head injury, and also in biopsies of human contused neural tissue (Yang et al.^1995^; McKeating and Andrews 1998).

*Janus Kinase/Signal Transducer and Activator of Transcription (JAK/STAT) Pathway* JAK/STAT pathway is the fundamental channel intended for transmission of growth factors and cytokines accountable for variety of biochemical processes including axon regeneration, inflammation, cell differentiation, proliferation, and death (Oliva et al. 2012). Activation of JAK-STAT pathway commenced with a particular ligand binding to receptor on cellular surface, which subsequently triggers internal transmission via JAK kinase recruitment. JAK triggers dimerization and expression of STAT components. Some STAT proteins are found in nucleus and regulate gene expression by binding to a specific DNA sequence. After TBI, inflammatory process decreases JAK/STAT expression, resulting in increased cell death in cortical pericontusional region (Oliva et al. 2012). After TBI, rat peri-injured cortex cells were treated with recombinant erythropoietin (rhEPO), which increased JAK2 and STAT3 phosphorylation and reduced apoptosis. The JAK2 inhibitor AG490 lowered pJAK2 and pSTAT3 levels while increasing mRNA expression of many apoptosis-associated genes, implying that JAK2-STAT3 pathway is activated (Zhao et al. 2011) (Table 1).

**Table 1:** Inflammatory mediators involved in secondary injury in TBI

Inflammatory mediators	Level with time of injury	Mechanisms/comments	References
Cytokines and chemokines			
Interleukin-1β	Within hours of a TBI, there is a rapid elevation. Peaks on days 1–2 and then drops off on days 2–4	Activating other proinflammatory pathways such as TNF-α	Hutchinson et al. (2007)
Interleukin-6	Peaks on day 1, decrease on days 2–3	Stimulate NGF production by astrocytes and post-traumatic tissue repair and aggravates blood–brain barrier function	Maas et al. (2010); Winter et al. (2002); Kossmann et al. (1996); Swartz et al. (2001); Winter (2004)
Interleukin-8	It stays raised for up to 4 days after an injury, with a peak on first day and a gradual fall on days 2 and 3	Promotes neutrophil infiltration and increases BBB dysfunction	Morganti-Kossmann et al. (1997); Whalen et al. (2000); Maier et al. (2001)
Interleukin-10	Levels rise quickly after TBI and stay high for several days before gradually declining	Through a variety of signalling channels, IL-10 promotes glial and neuronal cell survival and also reduction in inflammatory responses	Bell et a. (1997); Pinteaux et al. (2002); Basu et al. (2002), Stover et al. (2000); Tehranian et al. (2002)
Monocyte chemoattractant protein (MCP-1)	Peaks on first day, then drops and peaks by fourth day, but remains elevated until tenth day	Promotes macrophage infiltration	Rothwell (2003); Stirling (2004); Xu et al. (2004); Maier et al. (2005); Semple et al. (2009)
Transforming growth factor -β (TGF-β)	Peaks at day 1 and slowly decrease after 21 days	TGF-β plays a regulatory function in nerve regeneration by regulating immunological response, cellular activity, scar formation and neurite outgrowth	Morganti-Kossmann et al. (1999)
Tumour necrosis factor (TNF-α)	TNF-α expression increase within one hour following TBI, peak around 3 to 8 h and function normally within 24 h	Activation of microglia and astrocytes, influence blood brain barrier permeability, glutamatergic transmission and synaptic plasticity	Tuttolomondo et al. (2014)
Cellular mediators			
Astrocytes	Marker for reactive astrocytes (YKL-40)day 1 recorded an increase, while day 4 reaches at peak	Aggravate neuronal deterioration and transgenic depletion of reactive astrocyte	Myer et al. (2006); Bonneh-Barkay et al. (2010)
Microglia	Rapid elevation observed after 72 h of injury, Peaks at 3 months	Release of oxidative metabolites such as reactive oxygen, nitric oxide and nitrogen species) and pro-inflammatory cytokines (e.g. interleukin (IL)-1β, tumour necrosis factor-β (TNFβ) and interferon-γ (IFNγ)	Engel et al. (2000); Block and Hong (2005)
Triggers and brakes			
Adenosine	Increased within hours of damage, then dropped quickly after 12–24 h	Triggers neuroinflammatory responses such as activating microglia and triggering P_2_X_7_R-mediated inflammation	Bell et al. (1998); Davalos et al. (2005); Jassam et al. (2017); Liu et al. (2017a)
Complement	Peaks 1 day after injury, then declines during next 2–7 days	Promote neurogenesis and plasticity subsequent to brain injury	Kossmann et al. (1997)
Glutamate	Typically peak observed at day 1, decrease at days 2–3	Activate three different groups of metabotropic receptors	Chamoun et al. (2010); Niswender and Conn (2010)
HMGB1	Increase within 30 min of injury, Peak observed up to 72 h of injury	By interacting with RAGE and TLR4, it causes sterile inflammation and induces macrophages and endothelial cells to release TNF-α, IL-6, and IL-1	Li et al. (2017); Hammad et al. (2018)
Mitochondrial DNA	Peak day 1 and decline at day 3	Subsequent to cell death	Laird et al. (2013)

**Fig. 1:**
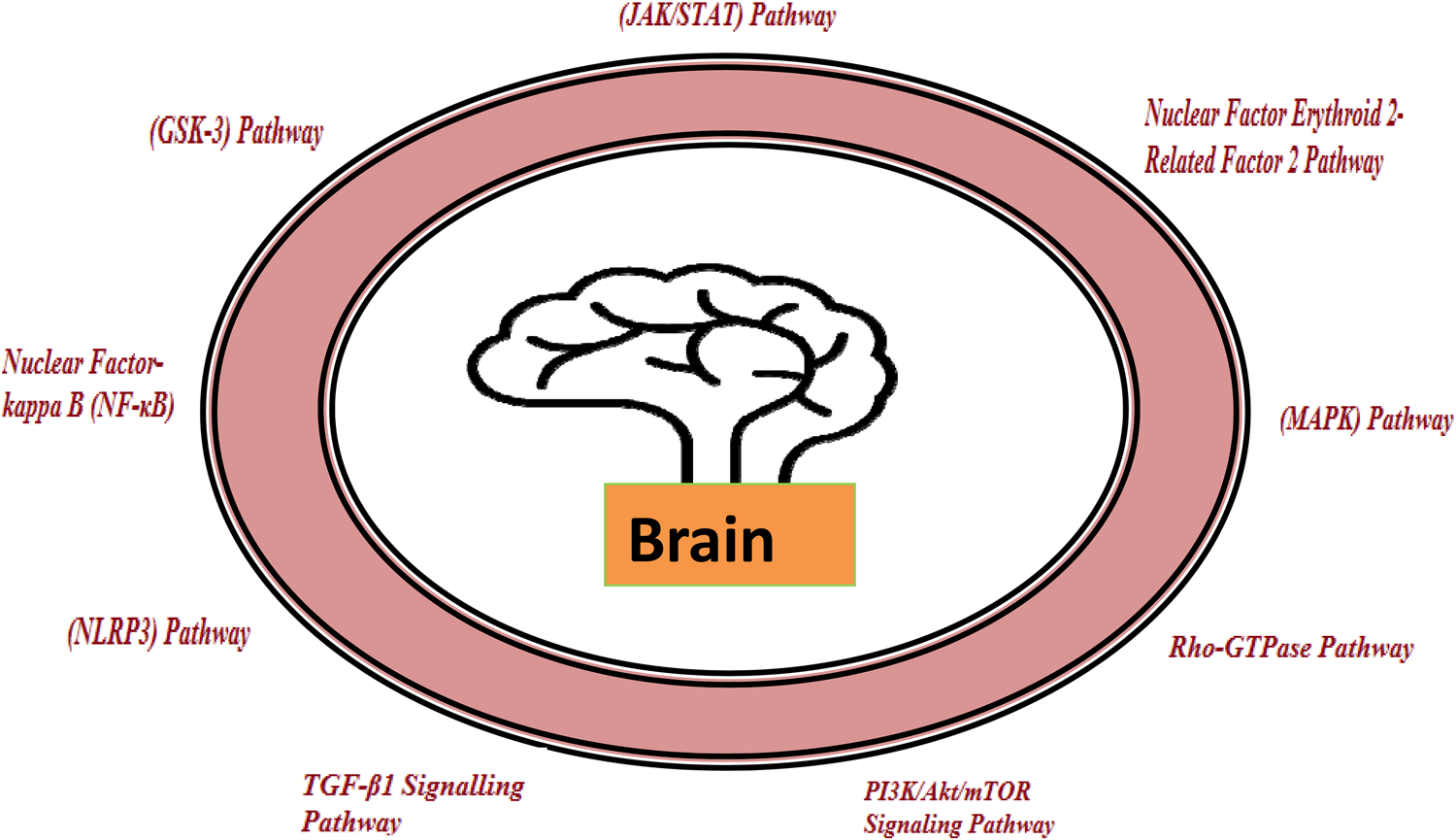
Various signalling pathways that affect the human brain (primarily neurons) in TBI

*Mitogen-Activated Protein Kinase (MAPK) Pathway* MAPK is a threonine/serine-dependent protein kinase which is triggered by phosphorylation in response to diverse cells trauma. It is important for cell differentiation, proliferation, and survival. Cascades are made up of c-Jun NH (2)-terminal kinase (JNK), extracellular signal-regulated protein kinase (ERK), and p38. Numerous studies reported that activation of p38 and JNK pathway elevates neuronal damage accompanying spinal cord injury and cerebral ischemia (Otani et al. 2002). Following TBI, cell line-based studies revealed astroglial growth and fast ERK activation (Carbonell and Mandell 2003). Pathophysiology of TBI includes abnormalities in the MAPK signalling pathway and studies revealed that blocking this cascade improved cell survival rate and significantly reduces intensity of cortical lesions. JNKs are stress stimulated protein kinases that are found in nucleus of neuronal cells and are linked to neurodegeneration. TBI can activate JNK and cause a complex cascade in mitochondria of brain cells, resulting in apoptosis (Chi et al. 2013; Dietrich and Bramlett 2016) (Fig. 1).

*PI3K/Akt/mTOR Signalling Pathway* During brain development, the PI3K/Akt/mTOR signalling cascade is an important regulator of neuronal cell proliferation, axon outgrowth, and dendritic formation (Kumar 2005). Various hormones and growth factors that affect mTOR complex and target molecules like mTORC1 and mTORC2 induce downstream Akt and activation of PI3K (Dibble and Cantley 2015). mTOR integrates input from various upstream signals to control cell death, cell growth inhibition, and autophagy. mTOR regulates synthesis of proteins in axons and cell bodies, which are necessary for cell development. TBI-associated symptoms like inflammatory reactions and epilepsy are mostly controlled by inhibiting mTOR pathway (Guo et al. 2013).

*Glycogen synthase kinase 3 (GSK-3) Pathway* GSK-3 controls protein synthesis, microtubule dynamics, glycogen metabolism, cell differentiation apoptosis, and cell death. Wnt and Akt are two important signalling pathways that regulate GSK-3β activity and are also known as protein kinase B (Fang et al. 2000). Due to Akt activation, phosphorylation of GSK-3β induces response of pro and anti-inflammatory in monocytes. Exclusive GSK-3βinhibitors provide protection to cells from proapoptotic stimulant depending on the role of GSK-3β in apoptosis. Irregular stimulation of GSK-3β is associated with chronic neuroinflammation and neurodegeneration. Several researches have shown that GSK-3β has a role in TBI models of neuroinflammation and also showed the potency of numerous inhibitors of GSK-3βin TBI (Li et al. 2014; Llorens-Marítin et al. 2014).

*Nuclear Factor Erythroid 2-Related Factor 2 Pathway* Nrf2 is a gene transcription component which protects cells from a variety of damaging stimuli. Nrf2 is mostly found in the cytoplasm, attached to its inhibitor Keap1, which restricts Nrf2 from entering the nucleus. In a recent study, it was found that Nrf2 downregulation promotes neuronal death and neuroinflammation by increasing oxidative stress, TGF-β1, NF-kB, and MMP3/9 (Suzuki and Yamamoto 2017).

*Rho-GTPase Pathway* Rho-GTPases (Cdc42, Rac1, and RhoA) are principal regulators of cell adhesion and cytoskeletal and cell adhesion controlling a wide order of cellular processes (Chi et al. 2013). Rho GTPase signalling dysregulation has been associated with aetiology of amyotrophic lateral sclerosis (ALS), Alzheimer’s disease, and Parkinson’s disease (Mulherkar and Tolias 2020). Continuous over expression of functional RhoA degrades neuritis repair and axonal regeneration following TBI, since it inhibits axonal regeneration as well as apoptotic responses. Astrocyte activation and proliferation are common responses to CNS damage (Mulherkar and Tolias 2020). The progressive formation of a scar-like structure by astrocytes, oligodendrocytes, microglia fibroblasts, and meningeal cells limits axonal regeneration and slows TBI recovery. In lateral fluid percussion injury model, RhoA activation has been observed in ipsilateral brain of rat (Dubreuil et al. 2006). Furthermore, it was also observed that RhoA activity was increased in neuroglia and spinal cord of rats and mice in rodent models of spinal cord injury models (Wu and Xu 2016).

*TGF-β1 Signalling Pathway* Microglial inactivation is characterized by TGF-β signalling. TGF-β1 has been shown to play a protective role in CNS diseases in previous research. Taylor et al*.* discovered that this pathway enhanced functional improvement after intracerebral haemorrhage by altering microglial cell alternative stimulation (Taylor et al. 2016).

*Nucleotide-binding domain (NOD)-like receptor protein 3 (NLRP3) Pathway* It is a multi-protein complex which aggregates and activates caspase-1 in response to hazardous stimuli and promotes release of the pro-inflammatory cytokines IL-1β and IL-18. Following TBI, immune system activated by these cytokines produces neuroinflammation that contributes to cell death (Lamkanfi et al. 2011).

### Anti-inflammatory drugs and TBI

Glucocorticoids and nonsteroidal anti-inflammatory drugs (NSAIDs) are primarily commercially available synthetic anti-inflammatory drugs. Glucocorticoids have potent immunosuppressive and anti-inflammatory properties. They suppress preliminary signs of inflammation like redness, discomfort, and swelling as well as the later stages of wound healing and its proliferative processes that occur in chronic inflammation. Indeed, steroids bind to certain intracellular receptors, forming a complex that later on modulates gene expression, inducing some proteins to be synthesized while others are inhibited (Barnes and Adcock 1993).Glucocorticoids also interact with AP-1, a heterodimer of Fos and Jun proteins that acts as a transcription factor activator. Inhibition of AP-1 inhibits the activity of leukocytes, lymphocytes, and mononuclear cells, as well as the release of pro-inflammatory cytokines including TNF-α and IL-2. Steroids also suppress COX-2 expression, a gene that is ordinarily activated by inflammatory mediators and produces inflammatory prostanoids. In addition, they prevent osteocalcin production in osteoblasts stimulated by vitamin D3. Also, steroids alter collagenase expression and diminish the production of lipocortin 1 (Barnes and Adcock 1993).

NSAID is a class of drugs having combined analgesic, antipyretic, and anti-inflammatory actions due to their inhibitory effect against cyclooxygenase (COX) enzyme. This COX inhibition remarkably reduces the level of IL-1β and also impedes IL-6 synthesis by modulating vasodilator prostaglandin synthesis pathways. Moreover, NSAIDs also inhibit calcium-dependent glutamate release which consequently attenuates glutamate-induced neurotoxicity Breitner 1996).

Most importantly, certain NSAIDs (e.g. aspirin, naproxen, ibuprofen, diclofenac, nabumetone, oxaprozin, and sulindac) also impede COX-1 enzyme causing unwanted adverse effects. However, novel compounds, on the other hand, work selectively on COX-2 and are thought to be more effective. Celecoxib and rofecoxib, two novel anti-inflammatory medicines, are designed to target inflammatory sites alongside protecting non-inflamed areas where COX-mediated prostaglandin generation may be advantageous (Leveugle and Fillit 1994; Lehmann et al. 1997; Ricote et al. 1998).

Another aspect of NSAIDs is that they stimulate the proliferator-activated receptor (PPAR), which causes transcriptional regulatory effects that decrease a variety of pro-inflammatory chemicals and microglial activity. Furthermore, several NSAIDs have antioxidant properties and inhibit NF-kB activation (Grilli et al. 1996) (Table 2).

**Table 2:** Anti-inflammatory drugs showing pharmacological potential in TBI

Sr. No	Drugs (Animal Models)	Dose	Outcomes of the study	References
1	Dexamethasone (Weight drop)	1 mg/kg, (i.p.) administered in rats after 5 min of injury	At days 1 and 2, but not at days 4 and 6, the expression of endothelial-monocyte activating polypeptide II, P2X4 receptor and allograft-inflammatory factor-1suppressed	Zhang et al. (2007)
2	Ibuprofen (Fluid percussion injury)	60 mg/kg, (i.p.) administered in mice after 5 min of injury	Cognitive function improved in NSS and rota rode at 24 h and 1 day of injury	Harrison et al. (2014)
3	Roficoxib (Fluid percussion injury)	10 mg/kg, (i.p.)administered in rats after 5 min of injury	Neuroprotective effect observed at 12–72 h of injury in hippocampus region of brain	Kunz et al. (2022)
4	Nimesulide (Weight drop)	6 mg/kg, (i.p.) administered in mice after 10 min of injury	Cognitive and motor function improved at 24 h after injury	Cernak et al. (2002)
5	Celecoxib (Controlled cortical impact)	50 mg/ kg, (p.o.) administered prior to injury in mice	IL-1 was inhibited, whereas the anti-inflammatory cytokine IL-10 was unchanged	Dash et al. (2000)
6	Carprofen (Weight drop)	5 mg/kg, (sc.) administered immediately after injury in mice	Inhibition of microglial activation, improvement in neurological function, induces cell proliferation and gliogenesis after TBI	Thau-Zuchman et al. (2012)
7	Meloxicam (Weight drop)	2 mg/kg, (i.p.) administered in rats after 30 min of injury	↓Brain oedema and lipid peroxidation	Hakan et al. (2010)
8	Etanercept (Fluid percussion injury)	5 mg/kg, (i.p.) administered in rats immediately after injury	↑Motor and neurological function, ↓ IL-6 and IL-1β level after 3 days of injury and ↓ TNF-α at both 3 and 7 days post-injury	Chi et al. (2013)
9	3,6′-dithiothalidomide (Weight drop)	28 mg/kg, (i.p.) administered in mice before 1 h of injury	Inhibit TNF-α synthesis and ↑cognitive function	Baratz et al. (2011)
10	Anakinra (Controlled cortical impact)	100 mg/kg, (i.p.) administered in rats after 2 h of injury	Decreases endogenous IL-1rn gene expression only for 24 h, with no impact following 72 h and 7 day	Anderson et al. (2013)
11	Etazolate (Weight drop)	10 mg/kg (i.p.) administered in mice after 2 h of injury	↓ Microglia, IL-1β, oedema and NSS	Siopi et al. (2013)
12	Salsalate (Controlled cortical impact)	50 mg/kg (i.p.) injected in mice after 30 min of injury and once daily for five consecutive days	↓ Activation of NF-κB, ↓ nitrite secretion by microglia and ↑cognitive function, expression of genes linked with neurogenesis and neuroprotection	Lagraoui et al. (2017)

### Anti-inflammatory herbal drugs and TBI

Both crude plant extracts and their isolated compounds have shown neuroprotective effects on nerve functions due to their anti-inflammatory and antioxidant properties. Plant extracts used in traditional system for the alleviation of pain, fever, and inflammation have found to contain several natural anti-inflammatory medicines. A few of these natural products’ processes have been partially explored in recent years, and they are currently considered for therapy of chronic inflammatory and neurodegenerative illnesses. The majority of these medicines work by suppressing COX-2 transcription instead of activity. They also inhibit the expression of a number of pro-inflammatory genes (Keshavarzi et al. 2019).

Recently, numbers of traditional supplements and herbal medicine have been studied in treatment of TBI. Both animal and cellular TBI models revealed elevated expression of NF-κB, TNF-α, IL-6, and IL-1.Treatment with osthol, a coumarin derivative derived from *Cnidium monnieri*, reduced inflammatory mediators and enhanced neurological functions alongside elevating the neuronal count surrounding the injured area. Furthermore, treatment with osthol lowered the production of numerous inflammatory mediators (Kong et al. 2019).

We evaluated previous study to analyse possible neuroprotective effect of several medicinal plants in brain injury, owing to the increasing number of research published in recent years. Medicinal plants included in Table 3 have shown pharmacological potential in different model of TBI.

**Table 3:** Anti-inflammatory herbal drugs showing pharmacological potential in TBI

Sr. No	Plant (phytoconstituent/extract)	Dose/Model	Mechanism	References
1	*Actaea racemosa*(Formononetin)	10 and 30 mg/kg Weight drop	↑ IL-10, ↓ IL-6 and TNF-α	Baez-Jurado et al. (2017)
2	*Artemisia annua* (Atesunate)	30 mg/kg Controlled cortical impact	↓ Inflammation, level of TNF-α, IL-1β, VEGF, BDNF, GDNF and iNOS	Gugliandolo et al. (2018)
3	*Cinnamomum zeylanicum* (Polyphenol E)	10 mg/kg Controlled cortical impact	↓ NF-κB, IL-6, IL-1, NCAM, Nrf2 and GFAP expressions	Yulug et al. (2018)
4	*Crocus sativus* (Crocin)	20 mg/kg Controlled cortical impact	↓ Activation of microglia, cell apoptosis, TNF-α and IL-1β	Wang et al. (2015)
5	*Panax ginseng*(Aqueous extract)	50, 100, and 200 mg/kg Weight drop	↓ Expression of TNF-α, MDA, AChE, nitrite and ↑ IL-6,SOD, GSH	Kumar et al. (2013)
6	*Malva sylvestris* (Methanolic extract)	250 and 500 mg/kg Controlled cortical impact	↓ Neuronal loss, synthesis of ROS, expression of TNF-α,IL-6, IL-1β, and LPO ↑ SOD	Qin et al. (2017)
7	*Salvia tomentosa* (Luteolin)	20 mg/kg Controlled cortical impact	↓TNF-α and IL-1β	Sawmiller et al. (2014)
8	*Dracaena cochinchinensis*(Aqueous extract)	40 and 80 mg/kg Weight drop	↓MDA, IL-1β, IL-6, TNF-α and cell apoptosis	Hu et al. (2018)
9	*Rosmarinus officinalis* (Aqueous extract)	40, 80, and 160 mg/mL Lateral fluid percussion	↓ ROS generation, GFAP-positive cells, level of IL-1β, TNF-α and IL-6	Gohil et al. (2010)
10	*Curcuma longa* (Curcumin)	75, 150, and 300 mg/kg Controlled cortical impact	↓ Cerebral oedema, level of AQP4 and IL-1β, activation of NF-κB ↑ neurological function	Momtazi et al. (2016)
11	*Curcuma zedoaria* (β-Elemene)	100 mg/kg Weight drop	↓ TNF-α, IL-1β, TLR-4, cell apoptosis ↑ neurological severity score	Samini et al. (2013)
12	*Drynaria fortune* (Aqueous extract)	20 mg/kg Controlled cortical impact	inhibited microglial/macrophage activation, ↓brain lesion volume and IL-6 ↑ IL-10, neurological severity score and cognitive function	Vosough-Ghanbari et al. (2010)
13	*Salvia miltiorrhiza* (Salvianolic acid)	25 mg/kg Controlled cortical impact	↓ TNF-α and IL-1β ↑ IL-10, TGF-β1 and neurological function	Singh et al. (2013)
14	*Satureja khuzistanica* (essential oil)	50, 100, and 200 mg/kg Weight drop	↓BBB permeability, intracranial pressure, neuronal cell death, level of TNF-α, IL-1β and IL-6 ↑ numbers of viable astrocyte and IL-10 level	Meng et al. (2018)
15	*Scutellaria baicalensis* (Baicalein)	30 mg/kg Controlled cortical impact	↓ degenerating neuronal count, TNFα, IL-6 and IL-1β expressions ↑ neurological functions	Wang et al. (2015)
16	*Cnidium monnieri* (Osthole)	10–40 mg/kg Weight drop	↓ inflammatory mediators, hippocampal neuron loss, cerebral oedema and ↑ neurological function, SOD, MDA, GSH Bcl-2/Bax and active caspase-3 level	He et al. (2012)

### Anti-inflammatory drugs and neuroprotective potential in TBI

Anti-inflammatory approaches to prevent and treat neurotoxicity-associated neurological diseases have proved effective in vast cell-based and pre-clinical models, although nothing has been confirmed in later stages of clinical evaluations. However, with conclusive experiments this therapeutic approach propounds encouraging prospects for clinical exploration. In experimental animal models of stroke, both glucocorticoids and general anaesthetic drugs have sparked a great attention in neuroprotection; however, this has yet to be proved in humans (Degos et al. 2022). At some stage of stroke, head trauma, and meningeal bleeding, glucocorticoids have been, however, reported to be ineffective. Classic hypnotics such as thiopental and midazolam have immune-modulating properties and can reduce inflammatory responses in the peripheral nervous system. In an experimental mouse model, they suppress chemotaxis, neutrophil adherence, and phagocytosis as well as impede the discharge of free radicals and pro-inflammatory cytokines; however, these activities have yet to be proven in humans. COX inhibitors (mainly nimesulide and indomethacin) exhibit neuroprotective activity in neonatal mice with brain lesions (Muller 2019). The communication repression between the brain and activated peripheral inflammatory cells through blood–brain barrier induces neuroprotective action. Moreover, COX inhibitors are also reported remarkably effective against depression and various other psychiatric disorders. Noteworthy, celecoxib has been affirmed as an effective drug to treat serious depression and schizophrenia, predominantly in the early stages. Furthermore, acetyl salicylic acid has demonstrated preventative and curative effect against schizophrenia (Degos et al. 2022; Muller 2019).

### Current status of anti-inflammatory drugs in management of TBI

Despite advancements in preventative, diagnostic, and surgical techniques, therapeutic choices have been limited for management of TBI. Till date, no pharmacological remedy has been found to provide neuroprotective effects by targeting secondary damage mechanisms (Lozano et al. 2015).

Rehabilitation therapy is used in the majority of the patients. Because damage caused by initial injury is nearly impossible to treat, the rational approach for therapy intervention that provides clinically relevant advantages is to prevent subsequent cell death. It provides a large treatment window due to a delay in harm caused by secondary cell death (Lozano et al. 2015). Targeting neuroinflammation among the secondary wave of biochemical pathways appealed to this extended time for intervention beginning. A variety of medications have been examined and reported to decrease inflammation in animals and TBI patients at the preclinical and clinical levels.

A systematic analysis was performed through US National Institutes of Health clinical trials database using various search strategy consisting of either single or combination of the following keywords: traumatic brain injury, anti-inflammatory drugs, COX-1, COX-2, and specific names of distinct anti-inflammatory drug such as aspirin, celecoxib, ibuprofen, diclofenac. However, no specific findings are obtained in relevance to intervention of anti-inflammatory drugs in prognosis of traumatic brain injury. Although the use of dexamethasone in prognosis of TBI patients with brain contusions and pericontusional oedema is under recruiting status of phase 3, hopefully it will give expected outcomes.

Some clinical evidences suggested certain clinically approved drugs having potential anti-inflammatory activity are used in the treatment of TBI and some are under clinical trials as mentioned in Table 4. Meta-analysis study by Begemann et al. (2020) affirms that TBI patients receiving progesterone, erythropoietin, or cyclosporine have higher chance of a favourable outcome comparatively to those receiving placebo.

**Table 4:** Anti-inflammatory drugs under clinical phases for TBI management

Sr. No	Drug	Type of study	Proposed mechanism	Status
1	Erythropoietin	Erythropoietin long term effect observed in patients which suffered from moderate to severe injury	Showed anti-inflammatory, antiapoptotic and anti-oedematous properties due to stimulation of JAK/STAT pathway	Phase III NCT03061565
2	Rosuvastatin	After TBI, rosuvastatin effects studied on cytokines	Modulates TNF-α, IL-1, and IL-6 to change the immune response following brain injury	Phase II completed NCT00990028
3	Progesterone	Progesterone for Treatment of TBI III (ProTECT)	Neuronal loss and cerebral oedema reduced, remyelination is improved, functional recovery is improved after progesterone infusion	Terminated at Phase III NCT00822900
4	Methylprednisolone	Infusion of Methylprednisolone for 24 or 48 h vs. Tirilazad for acute spinal cord injury	Suppress NF-kB activation and TNF-α expression	Phase III completed NCT00004759
5	Minocycline	Minocycline’s safety and efficacy in the treatment of TBI	IL-1β and Microglial activation reduced	Ongoing phase 1/II NCT01058395
6	N-acetyl cysteine	The safety and potential therapeutic efficacy in mild blast traumatic brain injury patients	Reduces neurological symptoms	Phase II NCT02791975
7	Anakinra	Study for moderate to severe TBI patients	Decrease in pro-inflammatory cytokines for first 48 h of injury	Phase II NCT02997371

### Challenges with anti-inflammatory drugs

Clinical trials including anti-inflammatory drugs have produced mixed outcomes so far. Nonselective COX inhibition, inadequate use of specific anti-inflammatory medicines for a given illness or illness progression/severity, sub-optimal dosing at specified location, or inadequate transmission through BBB to brain could all be reasons for varied outcomes (Gilgun-Sherki et al. 2006).

Long-term use of high-dose NSAIDs has been linked to the development of autoimmune disorders. The use of NSAIDs in TBI models is currently investigated; however, the results have been equivocal thus far. In a pre-clinical study, chronic ibuprofen treatment significantly increased cognitive and histological outcome. However, it failed to provide neuroprotection in a TBI model (Harrison et al. 2014).

Prolonged administration of ibuprofen in injured animals provides a much lower results as compared to placebo. Moreover, no significant differences in tissue atrophy level in hippocampus or cortex between treated and untreated mice were observed. These data imply that using high dosages of anti-inflammatory drugs for an extended length of time after trauma may diminish the neuroprotective effects of post-traumatic inflammatory cytokines (Harrison et al. 2014).

### Summary

Multiple studies in animal models of TBI have shown that neuroprotective therapies can reduce subsequent damage processes and/or enhance behavioural outcomes. However, none of these promising experimental neuroprotective treatments have been translated to improve clinical outcomes in human. As a result, the development of new anti-inflammatory medications for the treatment of neurodegenerative illnesses that are based on improved BBB transit and have a higher safety profile could result in beneficial therapy. Moreover, the heterogeneity of population genetics and the degree of pathology should be considered while developing novel anti-inflammatory medications. To truly understand the process and chemistry of anti-inflammatory drug delivery into the brain, more research is required. Hence, to decrease this native risk, more specific methods are required to modulate the inflammation. Inflammasome inhibition will be one approach. This technique, however, remains difficult due to a lack of or insufficient understanding of inflammasome structure and activation. Nonetheless, some research on psychiatric disorders has focused at inhibiting NLRP3, the best studied inflammasome. This antagonist endogenously formed after brain injury, and it reduces lesions size in animal models when administered systemically or intracerebrally. To conclude, neuroprotection strategies based on inflammation modulation must maintain the immunological defense and curative functions of inflammation along with eliminating its neurotoxic effects; as a result, three major anti-inflammatory approaches for the neuroprotection pathway can be evolved: modification of peripheral inflammation-CNS communication, modification of pro-inflammatory cytokines–intracerebral targets interaction, and modification of inflammasome production in brain cells.

## Data Availability

Information/data collected from open sources.

## Abbreviations

BBB: Blood brain barrier
BDNF: Bain-derived neurotrophic factor
COX: Cyclooxygenase
ERK: Extracellular signal-regulated protein kinase
GSK-3: Glycogen synthase kinase 3
IFN-γ: Interferon gamma
IL-1β: Interleukin 1beta
JAK/STAT: Janus kinase/signal transducer and activator of transcription
JNK: C-Jun NH (2)-terminal kinase
MAPK: Mitogen-activated protein kinase
MCP-1: Monocyte chemoattractant protein
NF-κB: Nuclear factor-kappa B
NLRP3: Nucleotide-binding domain (NOD)-like receptor protein 3
Nrf2: Nuclear factor erythroid 2-related factor 2
NSAIDs: Non-steroidal anti-inflammatory drugs
PI3K: Phosphatidylinositol-3-kinase
PPAR: Peroxisome proliferator-activated receptor
rhEPO: Recombinant human erythropoietin
TBI: Traumatic brain injury
TGF-β: Transforming growth factor-beta
TGF-β1: Transforming growth factor-beta1
TLR-4: Toll-like receptor 4
TNF-α: Tumour necrosis factor α
TRAF-6: Tumour necrosis factor receptor-associated factor 6
